# The Hospital Nursing Supplement

**Published:** 1895-10-12

**Authors:** 


### "The Hospital" Nursing Mirror.

Being the Extra Nursing Supplement of "The Hospital" Newspaper.

[Contributions for this Supplement should be addressed to the Editor, The Hospital, 428, Strand, London, W.C., and should have the word "Nursing" plainly written in left-hand top corner of the envelope.]

### News from the Nursing World.

### SIR EDWIN ARNOLD ON NURSES.

"India will absorb any amount of feminine ability and courage," said Sir Edwin Arnold in his eloquent address at the opening of St. Thomas's Medical School last week. He appealed to the men before him to encourage and help the women who studied medicine to fit them for a field of work in India which native prejudices debarred male doctors from entering. Sir Edwin said many kind things about nurses, and declared that he hesitated as to whether doctors or patients were to be most felicitated on the "professional female nurses" who faithfully and fearlessly carried out their duties to the sick. Quoting remarks made by a famous physician, Sir Edwin said he had told him of three most sovereign remedies. These were hot water, pure air, and good nurses. He spoke with keen appreciation of Miss Nightingale's work in the Crimea, and commented on "the instant and generous applause" with which the crowded audience received her name. The recital by Sir Edwin of his own poem on "Florence Nightingale" was acknowledged with great enthusiasm, and the address of the distinguished scholar will not easily be forgotten by hose who were privileged to hear it. As these verses are not published in a form easily accessible to our readers, we give them in another column.

### KHAMA AT THE LONDON HOSPITAL.

The visit of the African King Khama to the London Hospital on Monday gave great pleasure to the patients, in whose condition and surroundings he exhibited keen interest. In addition to spending two hours in the wards, he and his suite inspected the Nursing Home and the Medical College, and expressed themselves much gratified by the explanations (given through the interpreter) of all that they saw. Through the temporary closing of Shadwell Hospital, the London has at present to receive even more than its usual number of children, and to these small patients the visitors paid much attention.

### HELP WANTED FOR PLAISTOW.

Sister Katherine Twining has over fifty helpers engaged with her in nursing the sick poor at Plaistow, and there is more than enough for all of them to do. Besides maternity and district nursing there remains a vast amount of work which might be accomplished with no preliminary training. Any number of lay-helpers could find a field for their energies, and require no equipment beyond intelligent common sense, and a strong love for humanity. The various departments which district visitors fill in most parishes, are vacant at Plaistow, because the neighbourhood does not produce resident gentry or wealthy parishioners. But Sister Katherine has made practical arrangements for leisured ladies from other places to come and help her at Plaistow. She has a house now standing ready to receive voluntary workers, for such varying periods as they can spare from other duties. Each lady must contribute a fixed sum weekly towards the expense of the modest establishment, and by the new scheme not only will much-needed help be secured to Sister Katherine, but useful occupation will be provided for many good and enthusiastic women desirous of giving personal service to the destitute poor.

### CLEAN TOYS AND LIGHT BOOKS.

Books and toys are always acceptable gifts in hospitals, but a little care in their selection is certainly desirable. Patients are easily wearied with volumes that are heavy in either sense of the word, whilst small, indistinct type renders the best book in the world distasteful to the weak as well as to the poorly-educated. A constant supply of toys is needed for children of active minds, whose bodies are confined by accident or illness to the narrow limits of a hospital cot. The toys may be cheap and new or old and worn, but they ought at least to be clean. Alas! donors sometimes expect gratitude in return for books and toys which no doctor or nurse would sanction being admitted to any sick ward.

### GUY'S HOSPITAL TRAINING SCHOOL.

The surgical examination was successfully passed by the following probationers and lady pupils at Guy's Hospital: Bell, Busford, Britton, Cooper, Cooke, Davidson, Edmonds, Eaton, Huddleston, Harris, Hyland, Hinkley, C. Jones, Leir, Loveday, Miller, Morrah, Pye, Patterson, Powell, Roff, Salmon, Searle, Suarts, Tubbs, Thain, Traves, E. Taylor, Witherington, Woole, Weekes, Wooldridge.

### WOMEN GUARDIANS IN THE NORTH.

A meeting at Ulverstone of the Manchester Women Guardians' Association was well attended, Miss Grafton taking the chair. She spoke strongly of the necessity for women understanding the Poor Law, and Miss Mason afterwards read a paper on "Nursing in Workhouses," urging those present to help forward skilled nursing in workhouse infirmaries and to increase the number of nurses. Miss Stacey read a paper dealing with the care of feeble-minded women, and spoke in favour of homes for feeble-minded children.

### LIVERPOOL CHILDREN'S HOSPITAL.

The Hospital for Sick Children at Liverpool is a very homelike place. The out-patients' department, which is a very important one, is crowded daily with patients, who, whilst waiting, obtain wholesome refreshments at a very low rate. Cakes, milk, lemonade, and other drinks popular with the little ones are set out in a small room, which is gay with pictures and coloured texts. The wards are all upstairs, and the arrangement of cots in the middle as well as round the sides gives an overcrowded appearance. It certainly seems as if funds ought to be forthcoming in Liverpool to erect a children's hospital better proportioned to meet the requirements of so vast and liberal a city. A tiny mortuary, out of sight but conveniently placed, has received special attention, and the parents whose sickly children die in spite of skill and care (often sought too late), surely find death robbed of half its terror by the reverent care which surrounds it.

### NURSES FOR THE PORTSEA PAUPERS.

An economical proposal for supplementing the inadequate numbers of the nursing staff at Portsea Island Union was made at a recent board meeting by the Visiting Committee. They suggested, it is reported, that sixteen workhouse inmates of both sexes should be converted into paid assistants by means of weekly salaries, ranging from one to three shillings a-week. Either the Portsea Workhouse contains sixteen capable persons who ought not to be living on the rates, or else the Visiting Committee must think "anyone" is good enough to look after the sick poor. The chairman of the board wisely decided that such a measure should not be adopted without due consideration, and gave the casting vote in favour of a resolution to inquire into the procedure of other Guardians in such matters. It would be instructive to learn what position the shilling-a-week inmates would exactly fill in the sick wards when taken off the paupers' list in the manner suggested by the Visiting Committee.

### IRISH FEVER NURSES.

Rations, apartments, and eight pounds a-year form the remuneration offered to assistant nurses at both Tullow and Bagenalstown Fever Hospitals. If the Guardians secure nurses at that rate of pay, we wonder what they offer their scrubbers. The Longford Guardians recently decided to engage an assistant nurse at a salary of five pounds—if they can get her, should be added to the advertisement.

### TRAINING AT GLASGOW ROYAL INFIRMARY.

Before entering the wards of Glasgow Royal Infirmary as probationer-nurses, candidates must gain a certificate for the subjects comprised in the first course of education in nursing, including anatomy, physiology, hygiene, &c. The preliminary training, which has superseded the "month's trial," is reported to be eminently successful, and the well-known matron, Mrs. Strong, says that since the new system was introduced in January, 1894, only one probationer has left without completing her training, and there has not been a single instance of bad conduct. The infirmary no longer grants one year's experience to nurses for other institutions, but reserves its training for probationers on the staff.

### BRECHIN'S CHARITY DEMONSTRATION.

The demonstration recently carried out with marked success at Brechin was first proposed, we are told, by one who had had personal experience in his own family of the value of a Queen's Nurse. The people of Brechin took up the idea most cordially, and decided that the local branch of the Queen's Jubilee Institute should share with the Infirmary the proceeds of the demonstration. The procession, which paraded the town, was admirably managed and extremely interesting, and the directors of the Infirmary occupied one of the cars. The Ambulance Brigade was very popular, and exhibited stretchers, and gave examples of first aid. By a concert given in the evening £14 7s. 7d. was raised for the charities, and the demonstration resulted in the handsome additional sum of £85 12s. 5d.

### ACROSS THE CHANNEL.

"Are there no nurses in Paris?" is a not unnatural question from the Englishwoman who looked in vain for some familiar uniforms. Lay nurses now employed in most of the French hospitals are entirely clothed by the State, and the fashion of their working gowns is by no means attractive, for they are ill-fitting and often slovenly. The trim-looking, educated nurse of England is unknown in France, both dress and duties assigned to the women who there work in hospitals being in all respects of an inferior description to those demanded in our own land.

### DISTRICT NURSING IN DEVON.

The employment of trained district nurses is found to work most satisfactorily under the Devonshire branch of the Rural District Nursing Association. The report of Miss Sanders, the hon. secretary, shows that larger subscriptions are required to cover the expenses of training an increased number of nurses for fresh districts. Lady Poltimore is president of the branch, and there is an energetic committee. Subscriptions should be sent to the hon. secretary, at Stoke Hill, Exeter.

### NURSING IN HOMES.

Although Bradford citizens cannot claim immunity from sickness, and they agree individually with the doctors' opinion that a well-managed Nursing Home is a better place for "operation" cases than a small, crowded private house, yet they have hitherto been prejudiced against the neighbourhood of such an institution. When Miss Barton wanted to move her nursing home to larger premises it was only after the landlords of some fifty houses had objected to rent a house for such a purpose that she and another trained nurse bought the excellent house which they manage so successfully. After due attention to the sanitary arrangements, they had the rooms put into proper nursing order, and on each door a distinctive name painted. These give a clue to the previous careers of the owners, for Miss Barton, having earned an excellent certificate at "The London," has called a ward after her training school, and Miss Marshall, trained at St. Bartholomew's, has the title of her old hospital on another door. "Weir Mitchell" and "Freeman" are named in memory of training in massage and midwifery. Patients do great credit to the skill of the two nurses, and many Bradford citizens are now inclined to agree with the doctors that this home is an unmitigated blessing to classes for whom free hospitals are not intended.

### SHORT ITEMS.

We have been requested by the publishers to state that the "Popular Medical Monthly" is to be reorganised, and will shortly be issued as "The Nursing World and Hospital Record: a Journal for Medical Practitioners and Nurses."—The arrangements for the sick at Mountjoy Prison were condemned as "abominable" at an inquest held on a late prisoner, the jury considering the system of nursing highly defective.—*Nursing Notes* of this month contains some interesting articles on various subjects. A list of the books in the library of the Trained Nurses' Club also appears in its columns.—A window, subscribed for by the matron and nurses, has been placed in St. Michael and All Angels' Church at Wigan, to the memory of Esther Cook and Esther Lister, who died of typhoid at the Royal Albert Edward Infirmary.

### Elementary Physiology for Nurses.

By C. F. Marshall, late Surgical Registrar Hospital for Sick Children, Great Ormond Street.

### X.—THE DIGESTIVE SYSTEM.

The purposes of digestion are (1) to separate the nutritious from the non-nutritious matter in the food; (2) to dissolve the nutritious part so that it can enter the capillaries and be carried all over the body; (3) assimilation of the dissolved nutriment by the tissues.

### General Anatomy of the Digestive Organs.

The alimentary canal is a tube with muscular walls commencing at the mouth, connected with which are the lips, teeth, tongue, and palate, and next to which is the pharynx. Beyond the pharynx there is no control over the food, and we cannot help swallowing it when it reaches this point. The entrance to the windpipe is guarded by the epiglottis, which prevents food falling into the larynx when we swallow (for the food has to pass over this before it reaches the pharynx). After the pharynx comes the œsophagus, a tube leading to the stomach.

*The Stomach* is 10 or 12 inches long and 4 or 8 in diameter, and is divided into cardiac and pyloric ends, which we shall see have different functions. The stomach is a muscular bag lined by mucous membrane which secretes the gastric juice. At one end, the cardiac end, so called because it is close to the heart, the œsophagus opens into it; at the other, or pyloric end, it leads to the commencement of the intestines.

*The Intestines* consist of the duodenum, 10 to 12 inches long; the small intestine, 19 to 20 feet long; the large intestine, 5 to 6 feet in length; and the cæcum and vermiform appendix, 4 to 6 inches long. The whole of the intestine is a muscular tube lined with mucous membrane secreting digestive fluids at its upper part, the duodenum and small intestine, and terminating below in the rectum and anus, by which the fœces or waste parts of the food after digestion are passed away.

*The Digestive Glands.*—The general purpose of these is to manufacture the fluids by which digestion is effected. The simplest glands are those in the mucous membrane of the stomach and intestines; they are simple pits, single or branched. More complex glands are the salivary glands; the parotid situated between the ear and the jaw; the submaxillary and sublingual under the jaw and tongue respectively.

*The Liver* is a large and compact gland weighing about three pounds. It forms a fluid—the bile—which enters the duodenum, or first part of the small intestine, through the bile duct. Connected with the bile duct is the gall bladder, which is a kind of reservoir holding the bile.

*The Pancreas*, or "sweetbread," lies in a loop of intestines formed by the duodenum. The duct conveying its digestive juice joins the bile duct just before the latter reaches the duodenum.

### Classification of Food Stuffs.

These, considered chemically, may be referred to four chief heads. The various parts of the body consist of carbon, hydrogen, oxygen, and nitrogen, combined in various proportions, and forming very complex structures, together with certain salts—potash, soda, and lime, &c.—and water. Water makes 57 per cent. of the weight of a man. These complex bodies are further divided, according to the presence or absence of nitrogen, into nitrogenous and non-nitrogenous. The various food stuffs are :—

1. *Proteids*, consisting of carbon, hydrogen, oxygen, and nitrogen. These are contained in the lean part of meat, the white of egg, the gluten of flour, and the casein of milk.

2. *Fats or Oils, or Hydrocarbons.*—These have more hydrogen than is required to combine with oxygen to form water. They comprise all the animal and vegetable fatty matters and oils, *e.g.*, butter.

3. *Amyloids or Carbohydrates.*—These comprise the sugars and starches. They are contained in rice, oatmeal, arrowroot, potatoes, &c.

4. *Minerals.*—These consist of various salts, and are especially common in green vegetables.

The first thing to note is that the proteids are absolutely essential, for the body is constantly wasting, and one of the chief waste products is urea, which contains nitrogen. Therefore proteids must be supplied to replenish the constant loss of nitrogen. Otherwise a man will live on his own body, and he cannot live on fats and amyloids alone.

Proteids contain both carbon, hydrogen, and oxygen, as well as nitrogen, and hence they alone can sustain life, but they are not economical. A man requires about 300 grains of nitrogen and 4,000 grains of carbon a day. Supposing he were fed on lean meat only, one pound would give him the 300 grains of nitrogen required, but four pounds would be necessary to give the 4,000 grains of carbon; hence he would have to digest about four times the necessary quantity of meat. It is far more economical to make up the other 3,000 grains of carbon by half a pound of fat or a pound of sugar. Most substances we use as food are mixed, and contain several kinds of food-stuffs. Thus butchers' meat contains a considerable quantity of fat, and bread contains a proteid—gluten—together with starch, sugar, and a trace of fat. The most economical combination is about two pounds of bread to three-quarters of a pound of meat per day.

### The Physiology of Digestion.

The food entering the mouth is first masticated by the teeth. It is next acted on by the saliva secreted by the salivary glands. This gland contains a substance, called ptyalin, which converts starch into sugar, and so renders it diffusible. Saliva has no action on proteids or fat. The food first enters the stomach and is acted on by the gastric juice. When the stomach is empty its mucous membrane is pale; the stimulation of food causes it to become red, by determination of the flow of blood to it. This causes the glands to become active and drops of gastric juice exude into the stomach. Contractions of the muscular walls cause the food to roll about and get mixed with the gastric juice.

### Minor Appointments.

Miss Louisa Dawson has been appointed Night Sister, after three years' training, in this infirmary.

Royal Albert Edward Infirmary, Wigan.—Miss Alice Clark has been made Assistant Matron at this infirmary, where she trained, and has since held successively the posts of Nurse and Sister.

Miss Mary Woodman, who was also trained at this infirmary, has been made Sister of the Gidlow Extension Ward. We congratulate all three nurses on their promotion, and wish them every success.

St. Pancras Convalescent Seaside Schools, Margate.—Miss Fanny Wynne, who was trained at St. Thomas's Hospital, and afterwards worked at the Mildmay Institution for twelve years, has been made Nurse-housekeeper at these schools.

Lambeth Infirmary.—Miss Amy Florence Kerr has been made Assistant Matron at this infirmary. She was trained at the Royal Infirmary, Liverpool, where she afterwards held the post of sister for six years. Miss Kerr has been lady superintendent of the Oldham Nursing Association since 1892, and holds excellent testimonials. We congratulate the Lambeth Guardians on having secured a lady with so much experience, and wish her every success in her new post.

### Bedford College for Women, London.

(Communicated.)

The inaugural lecture of the newly-instituted "Course of Scientific Instruction in Hygiene and Public Health" was delivered by Louis Parkes, Esq., M.D., D.P.H., on Saturday afternoon, October 5th. The Chairman of the Council introduced the lecturer with a few words contrasting the extended college curriculum of 1895-96 with that of 1849.

Dr. Parkes spoke of the great desirability of sound scientific training for women, as on that must to a great degree depend their success in the professional pursuits now being opened to them. Having regard to the fresh fields and possibilities of employment as hygiene lecturers, teachers in schools, inspectors of workshops, laundries, and factories where female labour is employed, the council of Bedford College have instituted this course, which aims at providing instruction in what may be called science in everyday life, viz., the theory (as against rule of thumb), principles, and rules for leading healthy lives, watchfulness as to the health of the home being pre-eminently woman's work. The lecturer drew attention to the Public Health Acts, and how greatly sanitary legislation, guided by the labours of Chadwick, Farr, and others had succeeded in lowering the death-rate, and in practically exterminating typhus fever. It is to the application of the laws of sanitation that people owe their immunity from ague, Asiatic cholera, and the greatly diminished prevalence of enteric fever. But the very excellence of the administrative machinery of sanitary inspection imposes a corresponding responsibility on the individual, who must not be content to leave all to public bodies. It is to the ignorance of the elementary principles of hygiene, the consequent lack of proper care and attention to damp and dirty houses, that must be attributed the high rate of infant mortality and the prevalence of phthisis; while the alcoholic tendencies of the age are encouraged both by unhygienic houses and the artificially restricted dietary of the artisan. It is not to be expected that working-class mothers should read pamphlets on hygiene, but such knowledge and practice of the same can gradually be brought into their lives by women who really understand hygienic science, and bring personal influence as well as wise sympathy with the conditions in which the poor live to bear upon the matter. County Councils have already recognised the importance of both hygiene and domestic economy, and scheduled both these as subjects which it is desirable to have taught. The course of instruction arranged at Bedford College includes hygiene, physiology, bacteriology (than which there is no science more important to the student of hygiene), physics, and chemistry.

A vote of thanks to the lecturer was moved by Mr. George Griffith, and seconded by Miss H. Busk, who quoted most appositely from a speech of Sir Spencer Wells at the York Congress in 1886. The proceedings closed with a few words from Miss Pycroft, who urged the necessity of women inspectors having a knowledge of chemistry.

### Royal British Nurses' Association.

We are asked by the Secretary of the Royal British Nurses' Association to state that a meeting of the General Council will be held at 17, Old Cavendish Street, S.W., on Friday, October 18th, at five p.m., and that the first sessional lecture of the season will be given by Dr. Louis Parkes, D.P.H., &c., on Friday, November 22nd, 1895, at eight p.m., on "The Importance of Breathing Fresh Air." Admission to members, free; to non-members, on payment of one shilling. It is hoped that a large audience will assemble to hear Dr. Parkes' valuable remarks.

### Florence Nightingale.

By Sir Edwin Arnold.

Recited by him at the Opening of St. Thomas's Medical School.

If on this verse of mine
Those eyes shall ever shine
Whereto sore wounded men have looked for life,
Think not that for a rhyme,
Nor yet to suit the time,
I name thy name, best victress in the strife;
But let it serve to say
That when we kneel to pray,
Prayers rise for thee thine ear can never know,
And that thy gentle, gentle deed
For heaven and for our need
Is in all hearts as deep as love can go.
'Tis good that thy name springs
From two of earth's fair things—
A stately city and a sweet-voiced bird.
'Tis well that in all homes
Where thy kind story comes,
And brave eyes fill, that pleasant sounds be heard.
Oh! voice, in night of fear,
Like night bird's—sweet to hear;
Oh! strong heart, set like a city on a hill;
Ah, watcher! worn and pale,
Dear Florence Nightingale;
We give thee thanks for thy good work and will.
England is glad of thee;
Christ, for thy charity,
Take thee to joy when h art and hand are still.

### "The Hospital" Convalescent Fund.

The hon. secretaries have received a donation of 10s., kindly sent by an anonymous policy-holder through the secretary of the R.N.P.F. The nurses of Mill Road Workhouse Infirmary, Liverpool, have contributed 12s., and set a praiseworthy example to other nursing staffs. The maintenance of a free bed for the use of delicate or tired nurses requiring change of air is an object which should appeal to all our readers.

### Notes and Queries.

### Queries.

(4) *Lectures.*—I should like to know of some lectures on nursing and hygiene put in an attractive form for country villages.—*Trained Nurse.*

(5) *October 5th.*—Please tell me whether there is any chance of my getting a copy of The Hospital for October 5th. I have been to many shops and stalls to-day (Monday), and am told they are all "sold out."—*Regular Reader.*

(6) *Secretary.*—Will you be so good as to tell me whether secretaries to charities are always paid by commission as well as salary? Also if you think it a good plan for a small association to secure the services of a secretary who is already engaged in another similar institution? Would his having this connection amongst charitable people be of special advantage?—*Ignoramus.*

(6) *American Journal.*—Will you kindly tell me where I can get "American contemporary, *The Trained Nurse,*" of which you spoke last week?—*Nurse M. P.*

### Answers.

(4) *Lectures (Trained Nurse).*—We fear from your question that you confuse the delivery of lectures with the practice of merely reading aloud. A trained nurse who lectures on hygiene and nursing must write out her own lectures, studying to make them accurate, interesting, and intelligible to the class of hearers for whom they are intended. Although she should prove to herself, by thus writing out her subject, that she knows thoroughly what she is going to teach, the lecture should be learned by heart. Notes to ensure that essential points are not omitted may, of course, be used. It is most desirable for the speaker to see that her audience comprehend her instructions, but if her eyes are fixed on a book she fails to respond to the puzzled look or inquiring glance which appeal for additional explanations. If "Trained Nurse" proposes to use other people's lectures she must call them "readings" on nursing, and give the author's name when she does so.

(5) *October 5 (Regular Reader).*—Apply at the office, 428, Strand.

(5) *Secretary (Ignoramus).*—A distinction must be drawn between the duties of a secretary and a collector, although they are sometimes combined in the same person. The latter is often paid by a commission, a small percentage being given on the whole sum collected, and a larger one on new subscriptions. A small institution may well share its secretary, but not with another charity having the same scope of work. A "connection" among charitable people is chiefly useful as regards the collector.

(6) *American Journal (Nurse M. P.)*—It can be obtained through the Scientific Press, 428, Strand, who have consented to supply it in England.

### A Visit to the Pasteur Institute.

By a Nurse.

"Is it your first visit, madame?" queried a nervous little Frenchwoman as I seated myself one hot summer morning in the shady garden at the Pasteur Institute.

"Yes, madame," I replied. "I have never seen this fine building before. Probably you are under treatment?"

She was evidently glad to be questioned, and in a few moments had told me her history. She was a workwoman, and had been bitten last week by a little pet dog.

"Madame understands, perhaps, that all the persons here have been bitten? Not by savage beasts; ah! no. Sometimes it was just a small affair; but then it was always safer to come for the inoculation, much safer!"

I hoped, in my best French, that madame was not much hurt, "the little dog was not unhealthy ?

"Ah! no, no! but the *patron* was a little nervous; and," with an expressive shrug of her little shoulders, "behold! here I am, for the third inoculation."

"Madame must go in and report, if it is her first visit," said a stout, red-faced man who stood near. "The operations take place at eleven o'clock precisely."

I explained that I had come, by permission, to look on only, I had no need to become a patient. This confession immediately deprived me of the man's attention, his interest in me ceased, and he turned away and talked volubly to a middle-aged acquaintance. He told particulars of his own accident, and then informed all within hearing that the little son who accompanied him was greatly inferior in bulk and size to himself at that age.

New-comers had an air of excited expectancy, but most who had been before were stolidly indifferent, and chatted together in little groups until eleven o'clock approached.

Then there was an adjournment to the waiting-hall, a pleasant, tiled room, well lighted and furnished with benches, and there were also dressing-rooms for men and for women.

I was invited to pass into another apartment where I sat with two doctors and a woman-student, strangers like myself, behind the operator's chair. On his left stood an assistant, who attended solely to the refilling of the little syringe, whilst the duties of another consisted in holding the patients firmly in front of the operator. This prevented the inoculation being rendered futile by a sudden movement or the involuntary retreat of the patient.

The new cases had two inoculations for the first five days they told me, and one for the ten succeeding days, so that the course of treatment for "bites" covered fifteen days. The doctor first took up a piece of cotton wool with his forceps, dipped it in some solution and rapidly passed it over the skin, and then as rapidly injected the desired quantity of fluid. The whole arrangements were very complete, and not a second of time was wasted during the whole performance.

Even the doctors were constrained to smile when it came to the turn of a very big German who towered several inches above the attendant who held him. Men, women, lads, and lassies succeeded each other swiftly, numbering in all 65; some days, I was told, nearly a hundred were done. A few of the young girls squealed shrilly when they saw the tiny syringe. Some small children had to be lifted up for the doctor to reach them, and he spoke to them very kindly. If they bore the prick of the little syringe bravely they were rewarded by *sous* from the doctor's pocket, and then retired wreathed in smiles.

On some faces there were a few tears and on others a look of anxiety, but the majority struck me as considering the treatment precautionary rather than necessary, and were quite content to submit to it.

The number of inoculations which it was customary to give and the quantity of fluid injected, were courteously explained by the assistant, before I followed the last patient from the room, and found the others already starting on their homeward journey.

"M. Pasteur is rather improved in health," I heard a hopeful voice murmur, and then I saw the laboratories and other rooms where he had prosecuted his researches.

The whole place was beautifully clean, and I followed my guide into the fine library, and tried to understand all his enthusiastic descriptions of the writers of the pamphlets scattered on the table. Such a beautiful room it looked in a student's eyes, and no one imagined, that day, how soon it would be required to serve as a mortuary chamber for the great M. Pasteur himself.

"Would Madame like to see the animals?"

Of course I said "Yes," and followed the three other strangers to the shed where the dogs were being fed on hot messes. It was an ill-smelling place, and we did not linger there long. Dogs and guinea-pigs are inoculated for tuberculosis, the guide told us, pigeons for typhoid, cocks and guinea-pigs for cholera, guinea-pigs for anthrax, rabbits for hydrophobia. The animals seemed neither well kept nor cleanly, and not one of us had a word to say in praise of their appearance.

H. F. G.

### Everybody's Opinion.

[Correspondence on all subjects is invited, but we cannot in any way be responsible for the opinions expressed by our correspondents. No communications can be entertained if the name and address of the correspondent is not given, or unless one side of the paper only be written on.]

### "FIRST AID."

"K. H. T." writes: May I through the pages of your valuable paper tender my sincere thanks to the lady who witnessed the recent accident at Conway, and who in such kindly terms mentioned my part in the occurrence in The Hospital of last week? I can only say how glad I was of the opportunity of rendering what assistance I could in such an emergency. How valuable the knowledge of "first aid" is amongst all classes! In this instance, however, no one seemed to have the least idea what to do.

### NURSES: THEIR UNIFORM AND THE PUBLIC.

"A Superintendent" writes: May an old nurse be allowed to thank you for your article on this subject, and to say that if the action of the hotel manager, who declined to allow a nurse to appear at table in her uniform, results in making nurses think twice before unnecessarily displaying a distinctive garb in public he will have earned the thanks of many who would gladly see outdoor uniforms suppressed. The fact that a nurse's dress is now so largely copied by persons who have no sort of right to wear it makes one wonder if the time has not arrived when the purpose for which this distinctive dress was thought necessary would be better served by its abolition. That district nurses must wear an outdoor uniform is obvious, but why any others need one is not by any means so clear. I trust that your article may lead to a helpful discussion of the subject, which has certainly now become an important one.

"Bicycle" writes: *Apropos* of "Nurses' Uniform and the Public," the editor's suggestion—more, conviction, that nurses should not wear their uniform except when on duty or about their usual avocations—would, I think, result unsatisfactorily. Every nurse knows what a dowdy she looks when she adapts her uniform to ordinary uses, and how unnurse-like she looks when she attempts to do *vice versa*. Neither our salaries nor our box accommodation would allow of nurses adopting two styles of dress with any nicety of appearance resulting. It is not for the true nurse to be ashamed of her uniform; on the contrary, she should, I think, wear it modestly on all occasions, not attempting any stylishness which might be misinterpreted. Of course, London nurses are a law unto themselves. A large majority of them are their own mistresses, and they can, and I have no doubt do, exhaust their purses in dress and sight-seeing to an extent which would astonish their thrifty country cousins or sisters. These I put out of court, and leave them to their jaunty little shoes, complexion veils, &c. Is it material to the subject under discussion whether or no they lunch with their relations at big hotels?

### A TRAINED AND CERTIFICATED VILLAGE NURSE.

"The Hon. Secretary and Superintendent of Ramsbury Nurse Association" writes: The subject of village nursing is so much to the fore in these days that it may interest some of your readers to learn what has been the experience of the District Nursing Association in the large agricultural parish of Ramsbury, Wilts. Started under the presidency of Lady Burdett, of Ramsbury Manor, just a year ago, the first balance-sheet (that crucial test), which has been lately circulated, is eminently satisfactory. The total expenditure for the twelve months has been £68 7s. 7d., which has been more than met by subscriptions, donations, extra fees for private nursing, and by the proceeds of the "Nurse Club," the members of which, numbering 100, pay 2d. per month, and are thus entitled to attendance in sickness. No charge is made to the very poorest. The committee were fortunate in securing the services of a very efficient and valuable trained and certificated nurse from the Brownlow Hill Infirmary, Liverpool, who has fully justified the high recommendations she brought with her, and who has in a marked degree gained the confidence and gratitude of her patients. She has paid 3,862 visits in the year, but the mere numerical statement says nothing of the brightness and relief nurse's daily visit brings into many lives of monotonous chronic suffering, nor does it tell of the patient, watchful skill shown by her in the more serious cases of illness which have occurred from time to time. So far we have reason to be well satisfied with our "nursing" experiment, and have good reason to hope that the work so well begun will go on and prosper.

### HOURS OF ASYLUM ATTENDANTS.

Dr. Urquhart writes from James Murray's Royal Asylum, Perth: With reference to the letter from an "Asylum Nurse" in your issue of the 5th inst., it appears to me necessary to make some protest against the statements therein contained. At a time when every effort is being made to improve the status of this class, the publication of such a letter is really detrimental. It may be admitted that the hours are nominally long, but in no properly-managed institution is a nurse subjected to the wear and tear of mind and body consequent upon sixteen hours' daily management of acutely excited cases, who, after all, constitute but a very small minority of the population of an asylum, and who are cared for by relays of nurses for short periods. Anyone who has had practical experience of hospital and asylum nursing knows that the fatigue of a day's work is not less in one than the other. I hazard the opinion from an "Asylum Nurse's" letter that she is a practitioner whose services are not likely to be long required. If an asylum nurse is doing her duty, she has nothing to fear from head attendants or their deputies. On the contrary, she will have every sympathy and encouragement. Those responsible for the management of asylums have to consider the patients in the first place, not forgetting their duty to the ratepayers as well as to the officials. From this practical point of view we must adhere to the hours found most suitable by experience, and the day staff must remain on duty practically from six o'clock in the morning till eight o'clock at night. But this does not express the entire truth, because, taking the staff as a whole, much of this time is spent out of doors at exercise, or in simple duties indoors, which cannot be termed exhausting. Of course, nurses are expected to work when there is work to be done, because, unless they lead the way, work will be but perfunctory. The tendency is to increase the staff of supernumeraries, so as to permit nurses to enjoy much-needed recreation and change at stated intervals and during annual holidays. The fact is, that the more incompetent the person the better opinion she has as to her qualifications for nursing; but a little experience in the wards soon rubs the bloom off that opinion—then follows a letter to The Hospital. Looking back over an experience of three-and-twenty years, one is encouraged by the great improvements already effected in asylum nursing to expect still better things. There is yet so much to accomplish, both possible and proper, that one is impatient of the spirit which dictates the kind of letter to which I have referred: and I am assured that it does not express the average opinion of the nursing world.

"A Nurse in a Private Asylum" writes: Having read with much interest the letter of an "Asylum Nurse" in your last issue, I shall be very glad if you will allow me a little space to add my "tale of woe," and state how heartily I agree with every word used by your able correspondent, for, being in a private asylum, I can endorse them most thoroughly, for from the time we go on duty, at half-past six, until we go to bed, at ten, eleven, or sometimes twelve o'clock, we have not one minute to call our own. We nurses—so-called (to my mind "general servants" would be a more fitting title; for, indeed, we one and all do the work of a "general")—besides having to wash and dress the patients, have to do housework of a most laborious kind. There are beds to be made, bed-rooms, dormitories, dining-rooms, and staircases to be swept and dusted; grates and windows to be cleaned; coals to be fetched in, and floors to be scrubbed and polished; besides other things too numerous to mention. This is all supposed to be done by eleven o'clock, and we nurses found spick and span in our uniform ready to take the patients out for an airing. In addition to this, we have to wait on them at table, mend their clothes, and bath them. If a patient attacks and hurts us, or tears our clothes, we are coolly told it is a part of our salary. Small wonder is it that when our longed-for "off duty" time comes, we are often too tired to do anything but lay down to rest. Even when we do get to bed we are not sure of being left in peace, for (and this to my mind is scandalous) we have to sleep in the patients' dormitories, and refractory patients' into the bargain, who are often very noisy and troublesome. Then, again, we may be called up at any minute by the night nurse to assist with a bad case. For our meals we are only allowed half-an-hour, and as each one has to cut for herself, besides getting it ready, each meal is taken in a scrambling and hasty fashion. These are the details of a nurse's life in a metropolitan asylum under a private doctor; I shall be very glad if they are given publicity.

### Appointments.

Port Said.—Lady Strangford Hospital.—Miss Longfield has been appointed Matron of the Lady Strangford Hospital at Port Said. She was trained at Chester General Infirmary, had private nursing experience in connection with that institution and with the North Stafford Nursing Institute, and was for three years matron of the Wallasey Cottage Hospital. We wish Miss Longfield every success.

Dawlish Cottage Hospital.—Miss Emily Young Palmer as been appointed Matron of this hospital. She was trained at the Devon and Exeter Hospital, where she was assistant nurse, and afterwards sister-in-charge of the operation-room and ward. Miss Palmer holds excellent testimonials from the medical staff, by whom her thirteen years of faithful work have been highly appreciated. We add our good wishes for her continued success to those of her fellow-workers, by whom her departure from the Devon and Exeter Hospital is much regretted.

